# Supplementary material for: Studies on U50,488H-induced increases in serum corticosterone level and diuresis in wildtype and mutant mice expressing phosphorylation-deficient kappa opioid receptor
Source: Adv Drug Alcohol Res. 2026 Oct 28;6:17289. doi: 10.3389/adar.2026.17289 (PMC13751541; doi:10.3389/adar.2026.17289)
Supplement: Supplementary file 1 [file Image1.pdf]

Supplementary Fig. S1

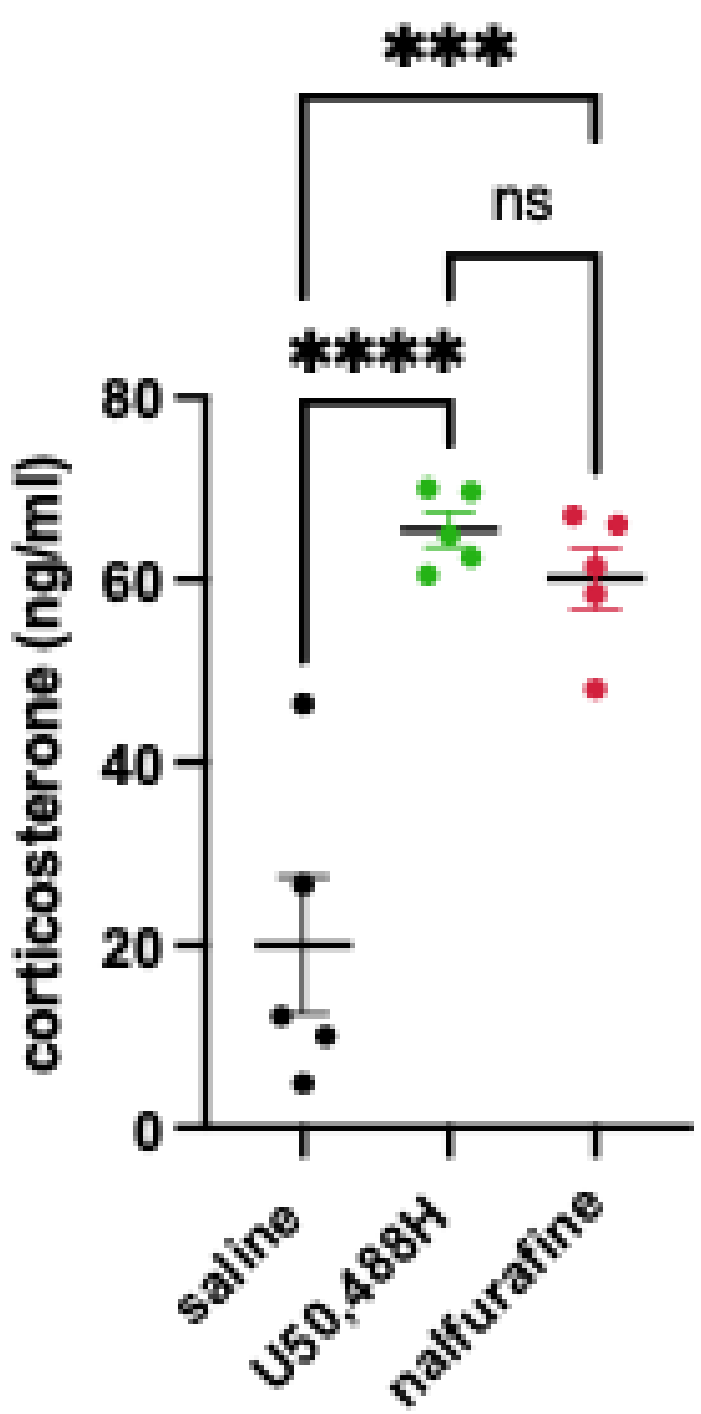

Supplementary Fig. S1 Effects of U50,488H and nalfurafine on serum corticosterone in mice.
Adult male CD-1 mice were treated with U50,488H (5 mg/kg, s.c.) or nalfurafine (20 μg/kg, s.c.). One hour later, blood was collected and serum was separated. Corticosterone levels in the serum were determined with ELISA. Results are expressed as mean ± SEM (n=5/group). Data were analyzed with one-way ANVOVA followed by Tukey's multiple comparisons test. ****P < 0.0001, ***p< 0.001, compared with the saline group. ns, not significant between the U50,488H and nalfurafine groups.
